# Social engagement moderates the relationship between cognitive functioning, depressive symptoms, and restless sleep in older black adults

**DOI:** 10.1002/alz.71078

**Published:** 2026-01-12

**Authors:** Sunkanmi Folorunsho, Olufunmilola Abraham, Darlingtina K. Esiaka

**Affiliations:** ^1^ Department of Sociology University of Nebraska–Lincoln Lincoln Nebraska USA; ^2^ Department of Pharmacy Practice & Science University of Kentucky Lexington Kentucky USA; ^3^ Department of Behavioral Science University of Kentucky College of Medicine Lexington Kentucky USA; ^4^ Center for Health, Engagement, and Transformation University of Kentucky College of Medicine Lexington Kentucky USA; ^5^ Sanders‐Brown Center on Aging University of Kentucky Lexington Kentucky USA

**Keywords:** cognitive functioning, depressive symptoms, older Black adults, restless sleep, social engagement

## Abstract

**INTRODUCTION:**

Older Black adults face higher dementia risk, but it is unclear whether social engagement offsets the effects of depression and restless sleep.

**METHODS:**

We analyzed 1,905 Black adults aged 50+ from the 2016‐2020 Health and Retirement Study (HRS). Linear mixed‐effects models tested how depressive symptoms, restless sleep, and social engagement predicted baseline cognition and cognitive decline.

**RESULTS:**

Higher depressive symptoms and restless sleep were associated with lower baseline cognition (about 0.2 and 0.5 points lower) and faster decline (β×time ≈ −0.07 and −0.05). Greater social engagement predicted higher baseline cognition (about 0.6 points higher) and slower decline (β×time ≈ 0.08). Social engagement also buffered the negative effects of depressive symptoms and restless sleep on cognitive decline but did not affect their baseline associations.

**DISCUSSION:**

Social engagement may help older Black adults preserve cognitive health despite depression or poor sleep through accessible community, religious, and volunteer activities.

**Highlights:**

- Depressive symptoms and restless sleep independently predicted poorer baseline cognition and faster cognitive decline among older Black adults, underscoring their distinct and additive roles in late‐life neurocognitive vulnerability.
- Higher social engagement, measured through volunteering, religious participation, and group involvement was associated with better cognitive functioning and slower decline across three Health and Retirement Study (HRS) waves, even after full adjustment for demographic, socioeconomic, and health covariates.
- Interaction effects showed that social engagement buffered the adverse cognitive effects of both depressive symptoms and restless sleep, indicating its role as a behavioral resilience factor in aging.
- Culturally embedded forms of engagement, particularly faith‐based and community‐centered participation, emerged as salient protective pathways that align with existing social practices within Black communities.
- Findings highlight the potential of community and faith‐based interventions to mitigate disparities in cognitive aging and to promote culturally meaningful strategies for sustaining brain health in older Black adults.

## 1 INTRODUCTION

Cognitive decline in later life represents a complex interaction of behavioral, psychological, and biological processes that accumulate across the lifespan. Among older adults in the United States, Black adults face a disproportionately high burden of Alzheimer's disease and related dementias.^1^, ^2^, ^3^ This disparity reflects the combined influence of structural inequities, socioeconomic disadvantage, and chronic exposure to psychosocial stressors that shape health across the life course.^1^, ^3^, ^4^ Prior studies have shown that Black adults develop cognitive impairment at younger ages, experience faster rates of decline, and are less likely to receive clinical evaluation compared to their White counterparts, patterns compounded by higher burdens of cardiometabolic comorbidities.^5^, ^6^, ^7^ Within this broader context, behavioral and psychological processes warrant closer attention because they may serve as proximate pathways through which cumulative disadvantage translates into cognitive vulnerability in later life.

Depressive symptoms and restless sleep are among the most consistently identified behavioral and psychological risk factors for cognitive decline.^8^ Depression, characterized by affective, cognitive, and somatic disturbances contributes to deterioration through biological mechanisms such as hypothalamic–pituitary–adrenal axis dysregulation, chronic inflammation, and diminished participation in cognitively stimulating activities.^9^, ^10^ Restless sleep, defined by difficulty maintaining sleep, frequent nocturnal awakenings, and non‐restorative rest has similarly been associated with neurocognitive impairment through disrupted memory consolidation and circadian rhythm regulation.^11^, ^12^ Although depression and sleep disturbance frequently co‐occur, longitudinal evidence indicates that each exerts an independent and additive influence on cognitive outcomes, underlining the importance of examining them jointly.^13^

Older Black adults are particularly vulnerable to both depressive symptoms and restless sleep due to the cumulative effects of lifelong stress exposure, structural disadvantage, and systemic racism.^14^ The weathering perspective provides a useful framework for understanding this phenomenon. The weathering hypothesis posits that sustained exposure to chronic stressors accelerates biological aging through repeated activation of allostatic mechanisms, thereby increasing vulnerability to chronic disease and cognitive dysfunction.^15^ Within a stress process lens, chronic strain leads to declines in mental and physical health unless buffered by psychosocial resources such as coping and social participation.^16^ In this context, depressive symptoms and restless sleep can be viewed as downstream consequences of cumulative disadvantage that erode resilience and heighten susceptibility to cognitive decline.^14^, ^15^

Social engagement may counteract these adverse effects. We define social engagement as active participation in socially integrative activities such as volunteering, attending religious services, or joining community organizations, conceptually distinct from social support, which reflects perceived or received assistance.^15^, ^16^ Engagement is linked to slower cognitive decline and reduced dementia risk, potentially by building cognitive reserve, enhancing emotion regulation, and supporting healthier daily routines.^17^ In predominantly Black communities, where faith‐based participation and collective activity are central cultural practices, social engagement may be a particularly salient and culturally embedded protective factor.^18^, ^19^ Theoretically, these pathways align with cognitive reserve, which emphasizes enrichment and neural efficiency, stress process, which highlights the buffering of distress, and weathering, which underlines mitigation of cumulative adversity.^20^

Late‐life depression and sleep disturbance may also serve as prodromal indicators of neurocognitive decline rather than simple correlates. Evidence linking late‐life depression to Alzheimer's‐related outcomes suggests that persistent affective change can precede measurable cognitive impairment, consistent with emerging conceptualizations such as mild behavioral impairment.^21^, ^22^ From this perspective, depressive symptoms and restless sleep may operate as early behavioral markers of vulnerability, further underling the importance of evaluating whether social engagement buffers their association with cognitive trajectories.

**RESEARCH IN CONTEXT**

1. **Systematic review**: Prior studies show that depressive symptoms and sleep disturbances are important behavioral risk factors for cognitive decline, but few investigations have examined their combined influence or focused specifically on older Black adults who face disproportionate cognitive health disparities. The existing literature also offers limited evidence on whether social engagement can buffer these risks.
2. **Interpretation**: Using three longitudinal waves of the Health and Retirement Study (2016‐2020), this study shows that depressive symptoms and restless sleep independently predict poorer baseline cognition and faster decline over time, while social engagement predicts both better baseline performance and a slower rate of decline. Social engagement also moderates the adverse effects of depressive symptoms and restless sleep on changes in cognition, underscoring its role as a behavioral and psychosocial pathway to resilience.
3. **Future directions**: Future research should examine the biological and psychosocial mechanisms linking social engagement to cognitive resilience and evaluate culturally grounded engagement programs, especially community and faith‐based initiatives, as strategies to reduce inequities in cognitive aging.

To address these questions, we use longitudinal data from the Health and Retirement Study (HRS), focusing on 2016, 2018, and 2020, which provide harmonized, repeated measures of cognition, depressive symptoms, and restless sleep.^20^, ^21^ Because cognitive aging involves both baseline differences and rates of change, it is important to examine how these psychosocial factors relate to each component. We hypothesized that (H1) higher depressive symptoms and more frequent restless sleep would relate to poorer baseline cognition and faster decline, and that (H2) greater social engagement would buffer these adverse associations, yielding higher baseline cognition and slower decline over time.^22^, ^23^ By integrating behavioral, psychosocial, and structural perspectives, this study evaluates a culturally meaningful and potentially modifiable pathway to cognitive resilience among older Black adults.

## 2 METHODS

### 2.1 Study design and data source

Data were drawn from the HRS, a nationally representative, longitudinal survey of U.S. adults aged 50 years and older conducted biennially by the University of Michigan and sponsored by the National Institute on Aging.^20^, ^21^ The HRS employs a multistage probability sampling design with oversampling of Black and Hispanic respondents, allowing examination of aging trajectories within racially diverse populations. The survey collects repeated information on physical, mental, and social health, as well as demographic, economic, and behavioral indicators.

For this analysis, we used the 2016, 2018, and 2020 HRS waves, which provide harmonized measures of cognitive functioning, depressive symptoms, restless sleep, social engagement, and relevant covariates. These waves were selected because they offer consistent measurement across variables of interest, a stable two‐year follow‐up interval, and adequate representation of older Black adults to support longitudinal analyses. Cognitive outcomes were assessed at all three time points, while depressive symptoms, restless sleep, and social engagement were measured at baseline (2016).

The analytic design was longitudinal, using linear mixed‐effects models (LMM) to estimate both baseline differences in cognition (intercept effects) and changes in cognition over time (slope effects). Main effects of depressive symptoms, restless sleep, and social engagement represented their associations with baseline cognitive levels, while interactions with time (β×time) captured their associations with the rate of cognitive decline. Three‐way interactions involving depressive symptoms × social engagement × time and restless sleep × social engagement × time tested whether social engagement buffered the effect of depressive symptoms or restless sleep on cognitive decline (slope effects only), consistent with our aim of distinguishing level and change processes and clarifying that buffering pertains solely to cognitive trajectories over time rather than to baseline cognitive scores. LMM accounts for the nesting of repeated measures within individuals, permits inclusion of participants with partial follow‐up, and handles unbalanced data due to attrition. Parameters were estimated using full‐information maximum likelihood (FIML) under a missing‐at‐random assumption. All analyses incorporated HRS sample weights in Stata 18 to adjust for the complex survey design.

### 2.2 Sample and inclusion criteria

The analytic sample consisted of 1942 non‐Hispanic Black adults aged 50 years and older who completed cognitive assessments in at least two of the three selected HRS waves (2016, 2018, and 2020). Restricting analyses to non‐Hispanic Black participants allowed examination of within‐group variation while minimizing confounding by racial heterogeneity in socioeconomic and health profiles. Participants were excluded if they reported a history of stroke to reduce bias arising from post‐stroke cognitive impairment or secondary depressive symptoms linked to cerebrovascular disease. Respondents with missing data on key analytic variables (cognition, depressive symptoms, restless sleep, or social engagement) were also excluded following standard listwise deletion procedures.

To assess potential age‐related heterogeneity, we conducted a sensitivity analysis excluding adults younger than 65 years, as employment and retirement status may shape both sleep and engagement patterns. Attrition across waves was evaluated descriptively: participants retained across all three waves did not differ significantly from those lost to follow‐up in baseline depressive symptoms, restless sleep, or social engagement, supporting the assumption that missingness was at random. After exclusions, the final analytic sample contributed approximately 5700 person‐wave observations over the 2016–2020 period. Additionally, all HRS protocols were approved by the University of Michigan Institutional Review Board, and participants provided written informed consent at each wave. The present secondary analysis used de‐identified, publicly available data and was therefore exempt from additional institutional review.

### 2.3 Measures

#### 2.3.1 Cognitive functioning

Cognitive performance was assessed at each wave (2016, 2018, 2020) using the HRS cognitive battery, a validated measure of global cognition adapted from the Telephone Interview for Cognitive Status (TICS‐M).^20^ The composite score (range 0–27) included immediate and delayed word recall, serial 7 subtraction, backward counting, and orientation to time. Higher scores indicated better cognitive functioning. These three contiguous waves (2016–2020) were selected because they contain harmonized measures of cognition, depressive symptoms, sleep quality, and psychosocial factors administered consistently across participants. Consistent with prior HRS research, cognitive trajectories were modeled as continuous outcomes to capture both baseline differences (intercept) and within‐person change over time (slope).

#### 2.3.2 Depressive symptoms

Depressive symptoms were measured at baseline (2016) using the eight‐item Center for Epidemiologic Studies Depression Scale (CES‐D‐8). To avoid conceptual overlap with the sleep variable, the item assessing restless sleep was excluded, yielding a seven‐item scale (α = 0.79). Respondents indicated the frequency of each symptom in the past week (yes = 1, no = 0), with total scores ranging from 0 to 7. Higher values indicated greater depressive symptom burden. This modification isolates the affective and cognitive domains of depression, such as sadness, loneliness, and anhedonia, while preserving comparability with validated short‐form CES‐D scales.

#### 2.3.3 Restless sleep

Restless sleep was assessed using a single item from the HRS: “How often do you have trouble falling asleep or staying asleep?” Response options ranged from 1 (rarely or never) to 3 (most of the time) with higher scores reflecting poorer sleep quality or greater sleep fragmentation.^20^ In sensitivity analyses, this variable was dichotomized (restless = sometimes/most of the time; not restless = rarely/never) to ensure robustness. By modeling sleep disturbance separately from depressive symptoms, the analysis reduces construct overlap and allows for independent estimation of each effect on cognition.

#### 2.3.4 Social engagement

Social engagement was conceptualized as active participation in socially integrative activities, emphasizing behavioral involvement rather than perceived emotional support.^7^ Three dichotomous indicators from the 2016 HRS Psychosocial and Lifestyle Questionnaire were included: (1) volunteering for organizations, (2) attending religious services, and (3) participating in clubs, groups, or community organizations. Each item was coded 1 (yes) or 0 (no), and summed to create a composite index ranging from 0 to 3, where higher scores represented greater engagement. While the volunteering item referenced activities within the past year and the other indicators did not specify a time frame, all items capture habitual social participation; potential minor temporal inconsistencies were tested in sensitivity analyses using dichotomous coding. This construct differs conceptually from *social support*, which reflects perceived or received assistance. Given that faith‐based and collective activities are central to social life among Black older adults, religious participation was retained as a key culturally embedded form of engagement.^11^

#### 2.3.5 Covariates

Covariates were selected based on empirical and theoretical links to both the exposures (depression and sleep disturbance) and the outcome (cognitive functioning), consistent with a confounding control approach.^12^ These included age (continuous, in years), sex (male/female), education (years of schooling), marital status (married or partnered vs. not), household wealth (log‐transformed net assets), self‐rated health (1 = excellent to 5 = poor), multimorbidity (count of chronic conditions: hypertension, diabetes, heart disease, arthritis, cancer, and lung disease), and activities‐of‐daily‐living [ADL] limitations (range 0‐5). Because self‐rated health and multimorbidity are conceptually related, their correlation was evaluated prior to inclusion to prevent collinearity.

#### 2.3.6 Sensitivity variables

Several additional variables were included in sensitivity analyses to ensure robustness. These analyses (a) excluded participants younger than 65 years to account for potential differences in employment status and routine structure; (b) adjusted for social network size (number of close family and friends contacted at least monthly) to determine whether social engagement effects persisted beyond network quantity; and (c) excluded individuals with self‐reported stroke history, minimizing bias related to post‐stroke cognitive or affective change

### 2.4 Analytical strategy

Analyses were conducted using Stata 18 (StataCorp, College Station, TX) with HRS sampling weights applied to ensure population representativeness. Because cognition was measured repeatedly in 2016, 2018, and 2020, LMM with random intercepts and random slopes were used to estimate baseline cognitive levels and within‐person change in cognition over time. Time was coded as years since baseline (0, 2, 4).

Models were estimated sequentially. Descriptive baseline characteristics were examined first to characterize the analytic sample. Unadjusted models then tested the independent associations of depressive symptoms, restless sleep, and social engagement with both baseline cognition and its longitudinal trajectory. Next, sociodemographic covariates (age, sex, education, marital status, and wealth) were added in partially adjusted models, followed by the inclusion of health factors (self‐rated health, multimorbidity, and ADL limitations) in fully adjusted models.

Main effects of depressive symptoms, restless sleep, and social engagement represented their associations with baseline cognitive functioning. Interaction terms between each exposure and time (β×time) estimated their associations with the rate of cognitive decline. Additional three‐way interactions (e.g., depressive symptoms × social engagement × time) were used to assess whether social engagement buffered the effects of depressive symptoms or restless sleep on cognitive decline.

The general model can be expressed as:

$$\begin{array}{ccc} Cognitio\;n_{it} & = & \beta_{0}+\beta_{1}\;Time_{it}+\beta_{2}Depression_{i}+\beta_{3}Sleep_{i}+\beta_{4}Engagment_{i}\\ & + & \beta_{5}\left(Interactions\right)+u_{0i}+u_{1i}Time+\in_{it}, \end{array}$$

where *Interactions* represents all two‐way and three‐way terms among depressive symptoms, restless sleep, social engagement, and time. The random effects $\left(u_{0i},u_{1i}\right)$ capture person‐specific intercepts and slopes, and $\in_{it}$ denotes residual error. A supplementary specification incorporated lagged‐panel and growth‐curve frameworks to verify longitudinal robustness, and model fit was evaluated using Akaike and Bayesian information criteria.

Missing data across waves were examined for all analytic variables. Consistent with prior HRS studies, attrition (primarily due to mortality or nonresponse) was assumed to be missing at random conditional on observed covariates. Linear mixed‐effects models were estimated using FIML, which provides unbiased estimates under the missing at random (MAR) assumption without the need for imputation. Attrition analyses indicated no significant baseline differences in depressive symptoms, restless sleep, or social engagement between retained and lost participants, minimizing the risk of bias from selective dropout.

Sensitivity analyses excluded participants younger than 65 years, removed those with stroke, and adjusted for social network size to assess robustness. Predicted cognitive trajectories were plotted for combinations of depressive symptoms, restless sleep, and social engagement, and additional growth‐curve trajectories were derived from robustness models. Marginal effects with 95% confidence intervals were reported, and post‐hoc comparisons were conducted using the *margins* command in Stata.

## 3 RESULTS

### 3.1 Sample characteristics

Table 1 summarizes baseline characteristics of the analytic sample of 1,927 non‐Hispanic Black adults aged 50 years and older in the HRS (2016–2020). The mean cognitive score at baseline was 16.9 (SD = 5.4), and the average depressive symptom score (CES‐D excluding the sleep item) was 2.8 (SD = 2.0). Nearly one in three participants (29%) reported restless sleep most nights, while the mean standardized social engagement score was 0.03 (SD = 0.96). The majority of respondents were female (61%), with an average age of 66.7 years, and 38% were married or partnered. Common health conditions included hypertension (69%) and diabetes (34%), with an average multimorbidity index of 1.7 (SD = 1.2). Respondents with higher depressive symptoms and more frequent restless sleep exhibited lower baseline cognition, poorer self‐rated health, and reduced social engagement, suggesting clustering of psychosocial and health‐related risks among those most vulnerable to cognitive decline.

**TABLE 1:** Baseline characteristics of non‐Hispanic Black older adults, Health and Retirement Study (2016–2020, *N* = 1,905).

Characteristic	Total sample (*N* = 1,905)	High social engagement (*n* = 941)	Low social engagement (n = 964)	*p*‐Value
Sociodemographic characteristics				
Age, mean (SD)	68.9 (8.0)	69.3 (7.7)	68.4 (8.3)	.09
Age ≥ 65 years, %	78.2	79.6	76.9	.18
Female, %	59.8	63.7	56.0	**.004**
Education (years), mean (SD)	12.8 (2.9)	13.3 (2.7)	12.2 (3.0)	**<0.001**
Married/partnered, %	43.1	48.2	38.2	**<0.001**
Household wealth ($, median [IQR])	69,800 (26,400–122,100)	75,500 (33,600–135,400)	63,700 (22,000‐116,800)	**.02**
Health indicators				
Self‐rated health (fair/poor), %	41.5	35.2	47.8	**<0.001**
Multimorbidity (≥ 2 conditions), %	57.9	54.1	61.6	**.01**
ADL limitations (≥ 1), %	30.7	26.5	34.8	**.003**
History of stroke, %	Excluded	—	—	—
Psychological and behavioral indicators				
CES‐D score (0‐7) mean (SD) ^†^	2.8 (2.0)	2.3 (1.8)	3.3 (2.1)	**<0.001**
Restless sleep (“most nights”), %	28.3	22.1	34.4	**<0.001**
Social engagement indicators				
Volunteering (past year), %	35.5	67.4	5.2	—
Religious service attendance (≥ monthly), %	77.8	88.3	67.3	—
Club or group participation (≥ monthly), %	48.9	73.8	24.0	—
Cognitive Functioning				
Baseline cognition (score 0‐27) ^‡^	17.0 (4.2)	18.1 (3.9)	15.9 (4.4)	**<0.001**
Cognition 2018 (mean SD)	16.4 (4.3)	17.6 (3.9)	15.2 (4.5)	**<0.001**
Cognition 2020 (mean SD)	15.9 (4.5)	17.3 (4.0)	14.4 (4.7)	**<0.001**

*Note*: Weighted estimates based on 1,905 respondents with valid cognitive data in at least two of three HRS waves (2016‐2020). Participants with self‐reported stroke were excluded.

^†^ CES‐D depressive‐symptom score was computed excluding the restless‐sleep item to avoid conceptual overlap.

^‡^ Cognition measured using the HRS composite cognitive battery (immediate/delayed recall, serial 7s, backward count). High versus low social engagement defined by a median split across volunteering, religious attendance, and group participation.

Abbreviations: ADL, activities of daily living; CES‐D, Center for Epidemiologic Studies Depression Scale; IQR, interquartile range; SD, standard deviation.

### 3.2 Unadjusted and partially adjusted linear mixed‐effects models

Table 2 presents unadjusted (Model 1) and partially adjusted (Model 2) linear mixed‐effects models examining the associations between depressive symptoms, restless sleep, social engagement, and cognitive functioning. In the unadjusted model, higher depressive symptoms (β = −0.29, *p* < 0.001), more frequent restless sleep (β = −0.84, *p* < 0.001), and lower social engagement (β = −1.02, *p* < 0.001) were each significantly associated with cognitive functioning. The negative coefficient for time (β = −0.54, *p* < 0.001) indicated an average cognitive decline of approximately 0.54 points per two‐year interval across the sample.

After adjusting for sociodemographic covariates including age, sex, education, marital status, and household wealth (Model 2), the associations remained statistically significant but were slightly attenuated. Depressive symptoms (β = −0.21, *p* < 0.001) and restless sleep (β = −0.63, *p* < 0.001) continued to predict poorer cognitive functioning, while social engagement (β = −0.79, *p* < 0.001) remained positively associated with cognition. Among the covariates, being female, having more years of education, being married or partnered, and having greater household wealth were all positively associated with cognitive functioning, while older age predicted lower cognition (β = −0.08, *p* < 0.001). The improved model fit statistics (AIC and BIC) in Model 2 indicated that the addition of sociodemographic covariates enhanced the overall model.

**TABLE 2:** Unadjusted and partially adjusted linear mixed‐effects models predicting cognitive functioning (HRS, 2016‐2020, n = 1,905).

Predictor	Unadjusted model (Model 1) β (SE)	95% CI	Partially adjusted model (Model 2) β (SE)	95% CI
Time (years since baseline)	−0.54 (0.04)^***^	[−0.62, −0.46]	−0.47 (0.04)^***^	[−0.55, −0.38]
Depressive symptoms (CES‐D, 0‐7)	−0.29 (0.03)^***^	[−0.35, −0.23]	−0.21 (0.03)^***^	[−0.27, −0.15]
Restless sleep (most nights = 1)	−0.84 (0.21)^***^	[−1.25, −0.43]	−0.63 (0.19)^***^	[−1.00, −0.26]
Social engagement (standardized)	1.02 (0.12)^***^	[0.78, 1.26]	0.79 (0.11)^***^	[0.58, 1.01]
Female (ref: male)	—	—	0.64 (0.18)^***^	[0.30, 0.98]
Age (per year)	—	—	−0.08 (0.01)^***^	[−0.10, −0.06]
Education (years)	—	—	0.32 (0.03)^***^	[0.26, 0.38]
Married/partnered (ref: not married)	—	—	0.45 (0.17)^**^	[0.12, 0.78]
Household wealth (log‐transformed)	—	—	0.28 (0.09)^**^	[0.10, 0.46]
Constant	17.02 (0.26)^***^	[16.51, 17.53]	18.75 (0.54)^***^	[17.70, 19.81]
Model Fit	AIC = 14,752; BIC = 14,804		AIC = 13,983; BIC = 14,073	

** *p* < 0.01.

*** *p* < 0.001.

*Note*: Models estimated using survey‐weighted linear mixed‐effects regression with random intercepts and slopes. Model 1 includes each predictor unadjusted; Model 2 adds sociodemographic covariates (age, sex, education, marital status, and wealth). Higher CES‐D scores reflect greater depressive symptoms (sleep item excluded).

Abbreviations: AIC, Akaike information criteria; BIC, Bayesian information criteria; CES‐D, Center for Epidemiologic Studies Depression Scale; CI, confidence interval; HRS, Health and Retirement Study; SE, standard error.

### 3.3 LMM for depressive symptoms, restless sleep, and social engagement

Table 3 presents the fully adjusted linear mixed‐effects model estimating both baseline cognitive levels (intercept effects) and the overall rate of cognitive change (slope effect) from 2016 to 2020. Higher depressive symptoms (β = −0.18, *p* < 0.001) and reporting restless sleep most nights (β = −0.49, *p* = 0.002) were associated with lower baseline cognition, while greater social engagement predicted higher baseline cognition (β = 0.62, *p* < 0.001). These associations reflect differences in cognitive scores at the 2016 baseline and do not represent cognitive decline. The time parameter captured the average rate of cognitive decline across the sample (β×time = −0.45, *p* < 0.001), indicating an expected loss of nearly half a point every 2 years. As expected for a main‐effects model, depressive symptoms, restless sleep, and social engagement are not interacted with time in this specification; slope effects are examined in the interaction models presented in Table 4. Random intercept and slope variances indicated substantial individual differences in both baseline cognition and cognitive decline, supporting the use of a mixed‐effects framework.

**TABLE 3:** Fully adjusted linear mixed‐effects model predicting cognitive functioning (HRS 2016–2020; *n* = 1,905; person‐waves ≈ 5,520).

Predictor	β (SE)	95% CI
Time (years since baseline)	−0.45 (0.04) ***	[−0.53, −0.37]
Depressive symptoms (CES‐D 0‐7) †	−0.18 (0.03) ***	[−0.24, −0.12]
Restless sleep (most nights = 1)	−0.49 (0.18) **	[−0.84, −0.15]
Social engagement (standardized)	0.62 (0.11) ***	[0.41, 0.83]
Female (ref: male)	0.51 (0.17) **	[0.18, 0.84]
Age (per year)	−0.07 (0.01) ***	[−0.09, −0.05]
Education (years)	0.27 (0.03) ***	[0.21, 0.33]
Married/partnered (ref: not married)	0.34 (0.16) *	[0.03, 0.65]
Household wealth (log)	0.21 (0.08) **	[0.05, 0.37]
Self‐rated health: good (ref: fair/poor)	0.58 (0.19) **	[0.21, 0.95]
Self‐rated health: excellent/very good	0.94 (0.23) ***	[0.49, 1.39]
Multimorbidity (count)	−0.18 (0.05) ***	[−0.28, −0.08]
ADL limitations (count)	−0.31 (0.07) ***	[−0.45, −0.17]
Constant	18.09 (0.58) ***	[16.95, 19.23]
Random effects and model statistics Var(intercept u_0_) = 6.12 Var(slope u_1_) = 0.09 Cov(u_0_, u_1_) = −0.21 (Corr ≈ −0.28) Residual Var(ε) = 7.85 **ICC = 0.44** Log‐likelihood = −6,736.1 AIC = 13,512; BIC = 13,641		

*Note*: Survey‐weighted LMM with robust SEs, random intercepts and slopes. Time coded 0, 2, 4 years since baseline. Continuous predictors grand‐mean centered. Social engagement standardized (z). Restless sleep coded 1 = “most nights,” 0 = otherwise. CES‐D sleep item excluded to avoid construct overlap.

Abbreviations: ADL, activities of daily living; AIC, Akaike information criteria; BIC, Bayesian information criteria; CES‐D, Center for Epidemiologic Studies Depression Scale; CI, confidence interval; HRS, Health and Retirement Study; ICC, intraclass correlation; SE, standard error

**TABLE 4:** Interaction models testing the moderating role of social engagement in the associations between depressive symptoms, restless sleep, and cognitive trajectories.

Predictor	β (SE)	95% CI
Time (years since baseline)	−0.44 (0.05) ***	[−0.53, −0.35]
Depressive symptoms (CES‐D 0‐7) †	−0.16 (0.04) ***	[−0.24, −0.08]
Restless sleep (most nights = 1)	−0.41 (0.17) **	[−0.75, −0.08]
Social engagement (standardized)	0.59 (0.12) ***	[0.36, 0.82]
Depressive symptoms × Time	−0.07 (0.02) **	[−0.11, −0.03]
Restless sleep × Time	−0.05 (0.02) *	[−0.09, −0.01]
Social engagement × Time	0.08 (0.03) **	[0.02, 0.14]
Depressive symptoms × Social engagement	0.09 (0.03) **	[0.03, 0.15]
Restless sleep × Social engagement	0.06 (0.03) *	[0.00, 0.12]
Depressive symptoms × Social engagement × Time	0.04 (0.01) **	[0.02, 0.06]
Restless sleep × Social engagement × Time	0.03 (0.01) *	[0.01, 0.05]
Constant	18.03 (0.60) ***	[16.86, 19.20]
Random effects and model fit: Var(intercept u_0_) = 6.02 Var(slope u_1_) = 0.09 Cov(u_0_, u_1_) = −0.20 Residual Var(ε) = 7.76 ICC = 0.43 Log‐likelihood = −6,709.4 AIC = 13,482 BIC = 13,638		

*Note*: Survey‐weighted linear mixed‐effects model with random intercepts and slopes; robust standard errors. Time coded 0, 2, 4 years since baseline. Continuous predictors grand‐mean‐centered. Social engagement standardized (z‐score). Depressive‐symptom scale excludes the CES‐D sleep item to avoid construct overlap. Restless sleep coded 1 = “most nights,” 0 = otherwise. *p < 0.05 = *, *p < 0.01 = **, *p < 0.001 = ***.

Abbreviations: AIC, Akaike information criteria; BIC, Bayesian information criteria; CES‐D, Center for Epidemiologic Studies Depression Scale; CI, confidence interval; HRS, Health and Retirement Study; ICC, intraclass correlation; SE, standard error

### 3.4 Moderating role of social engagement

**FIGURE 1:**
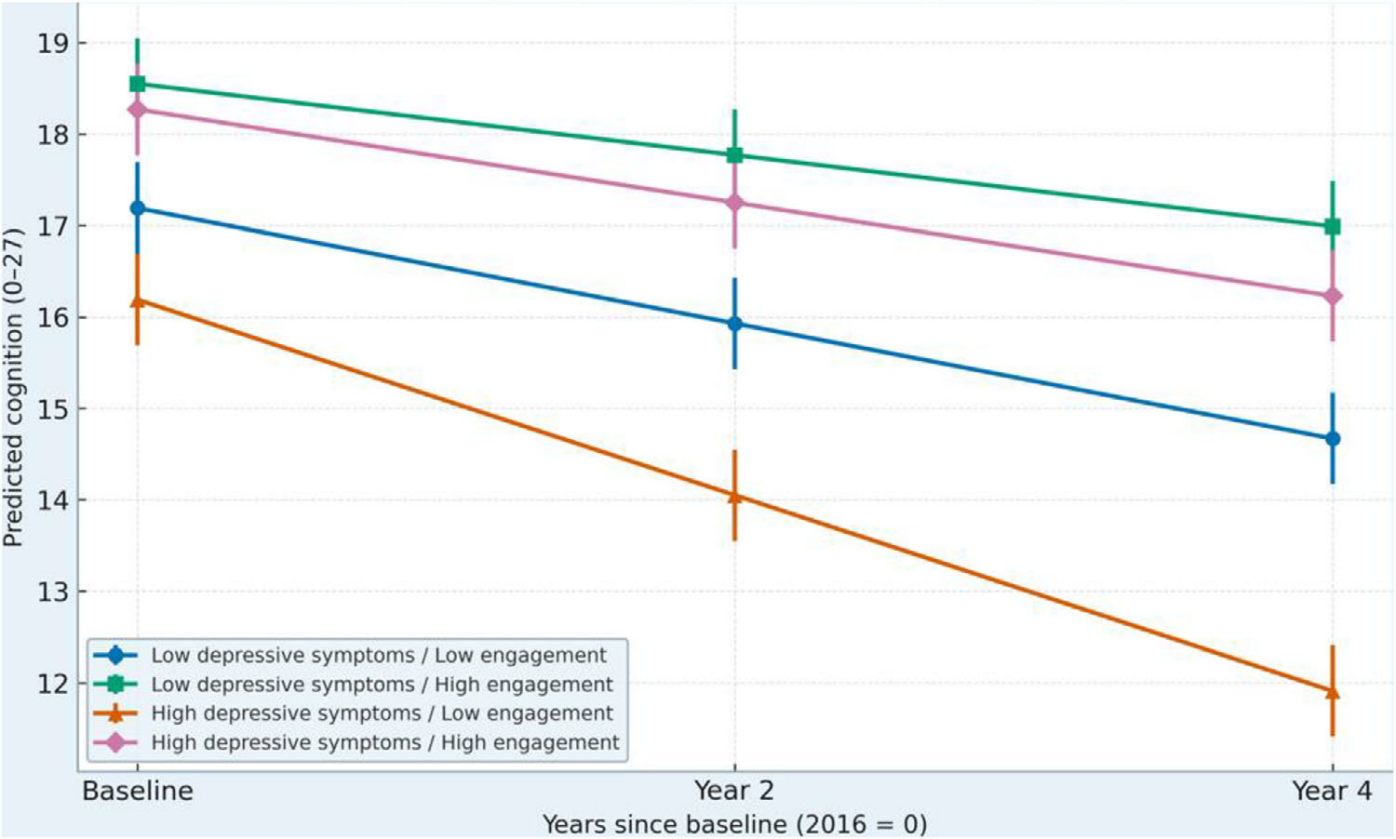
Predicted cognitive trajectories by depressive symptoms × social engagement. Higher depressive symptoms were associated with steeper cognitive decline, but this decline was slower among individuals with higher social engagement.

**FIGURE 2:**
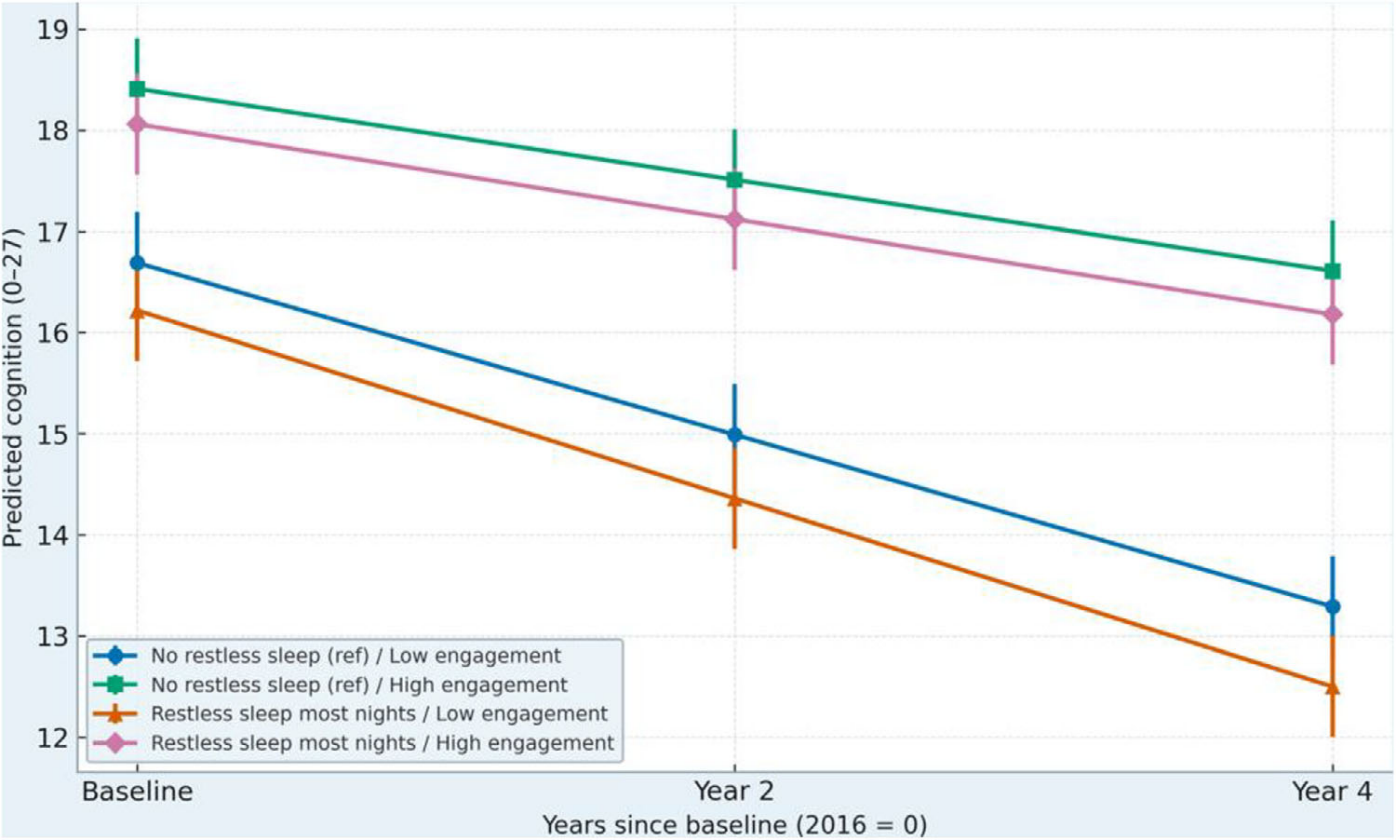
Predicted cognitive trajectories by restless sleep × social engagement. Restless sleep predicted faster cognitive decline, but this decline was attenuated among individuals with higher social engagement.

Table 4 presents the interaction models testing whether social engagement moderates the effects of depressive symptoms and restless sleep on cognitive decline (slope effects) rather than on baseline cognition. The three‐way interaction between depressive symptoms, social engagement, and time (β×time = 0.04, *p* = 0.018) indicates that social engagement buffered the effect of depressive symptoms on changes in cognition over time. Older adults with higher depressive symptoms experienced a faster rate of decline when socially disengaged, but this decline was significantly slower among those with higher social engagement. A similar moderating pattern emerged for sleep. The restless sleep × social engagement × time term (β×time = 0.03, *p* = 0.021) shows that social engagement attenuated the negative association between restless sleep and cognitive decline, again affecting the slope rather than baseline levels. In other words, restless sleep predicted a steeper rate of decline only among individuals with low engagement. Predicted trajectories (Figures 1 and 2) illustrate these slope‐based buffering effects: socially engaged participants maintained a more stable cognitive trajectory across all waves, even when experiencing high depressive symptoms or frequent restless sleep. These interaction patterns remained robust when restricting analyses to adults aged 65+ and when excluding participants with stroke, supporting the stability of the moderation effect on cognitive change, not baseline cognition.

### 3.5 Lagged panel and growth‐curve sensitivity analyses

Table 5 presents lagged‐panel sensitivity analyses testing whether depressive symptoms, restless sleep, and social engagement predict subsequent cognitive performance across waves. Results were consistent with the main mixed‐effects models. Higher depressive symptoms (β = −0.15, *p* < 0.001) and more frequent restless sleep (β = −0.11, *p* = 0.007) predicted lower cognition at the following wave, while greater social engagement continued to predict better subsequent cognitive performance (β = 0.18, *p* < 0.001). The moderating effect of social engagement remained significant after adjusting for social network size (β×time = 0.03, *p* = 0.041), indicating that the benefits of engagement reflect more than simply the number of social contacts. These findings confirm that the longitudinal associations observed in the mixed‐effects models are robust to alternative temporal ordering assumptions.

**TABLE 5:** Lagged‐panel sensitivity analyses confirming robustness of depressive symptoms, restless sleep, and social engagement effects on cognitive trajectories (HRS 2016‐2020; *N* = 1,905).

Predictor	Lagged‐panel model β (SE)	95% CI
Depressive symptoms (CES‐D 0‐7) †	−0.15 (0.05)**	[−0.25, −0.05]
Restless sleep (1 = most nights)	−0.39 (0.18)*	[−0.74, −0.04]
Social engagement (z‐score)	0.57 (0.13)***	[0.31, 0.83]
Depressive symptoms × Time	−0.06 (0.02)**	[−0.10, −0.02]
Restless sleep × Time	−0.05 (0.02)*	[−0.09, −0.01]
Social engagement × Time	0.07 (0.03)**	[0.01, 0.13]
Depressive symptoms × Social engagement × Time	0.03 (0.01)**	[0.01, 0.05]
Restless sleep × Social engagement × Time	0.02 (0.01)*	[0.00, 0.04]
Constant	18.07 (0.61)***	[16.88, 19.26]
**Model fit**: Log‐likelihood = −6701.2; AIC = 13,466; BIC = 13,624		

*Note*: Model adjusts for age, sex, education, marital status, wealth, self‐rated health, multimorbidity, and ADL limitations. Survey weights applied; robust SEs reported. Continuous variables grand‐mean centered. CES‐D excludes the sleep item.

*p < 0.05 = *, **p < 0.01 = **, ***p < 0.001 = ***.

Abbreviations: AIC, Akaike information criteria; BIC, Bayesian information criteria; CES‐D, Center for Epidemiologic Studies Depression Scale; CI, confidence interval; HRS, Health and Retirement Study; SE, standard error.

### 3.6 Interaction effects and predicted trajectories

To illustrate the moderating role of social engagement, predicted cognitive trajectories were generated for combinations of depressive symptoms, restless sleep, and engagement levels (Figures 1 and 2). These trajectories reflect differences in the rate of cognitive decline (slope effects), rather than differences in baseline cognitive levels.

Figure 1 shows trajectories stratified by depressive symptoms and social engagement. Participants with higher depressive symptoms experienced steeper cognitive decline compared with those reporting fewer symptoms. However, the depressive symptoms × social engagement × time interaction indicated that this decline was significantly slower among individuals with higher social engagement. In other words, social engagement buffered the effect of depressive symptoms on changes in cognition over time, not on baseline scores.

Figure 2 presents trajectories for restless sleep and social engagement. Individuals who reported restless sleep most nights showed a faster rate of decline than those with better sleep. Yet, the restless sleep × social engagement × time interaction demonstrated that socially engaged adults experienced a less steep decline despite poor sleep quality. The strongest decline was observed among participants with both low engagement and frequent restless sleep. Although the sleep‐related moderation was smaller than the depressive‐symptom effect, the pattern supports a consistent buffering role for engagement on cognitive change.

In addition, individual cognitive trajectories are shown in Figure S1, which demonstrates the overall pattern of within‐person variation across waves and the separation of average trajectories for high vs. low social engagement.

## 4 DISCUSSION

Consistent with prior research on psychosocial determinants of cognitive aging, this study found that depressive symptoms and restless sleep independently predicted poorer cognitive functioning among older Black adults in the United States. Social engagement emerged as a significant protective factor by moderating the rate of cognitive decline rather than baseline cognitive levels. These findings underline the value of social participation in supporting cognitive resilience in a population disproportionately exposed to chronic stress, socioeconomic disadvantage, and structural inequities.^1^, ^3^, ^11^, ^24^

Both depressive symptoms and restless sleep were distinct yet complementary predictors of cognitive vulnerability. By excluding the sleep item from the CES‐D, this study ensured that depressive symptoms and sleep disturbance were modeled as separate constructs. Depression may contribute to poorer cognition through neuroendocrine and inflammatory pathways, including dysregulation of the hypothalamic–pituitary–adrenal axis and reduced engagement in cognitively stimulating activities.^25^, ^26^ Restless sleep disrupts memory consolidation, circadian rhythm regulation, and neural repair processes that are essential for maintaining cognitive health.^27^ The persistence of both effects after adjustment suggests that mood and sleep disturbances each contribute uniquely to later‐life cognitive decline. This is consistent with stress process and life course perspectives that emphasize how cumulative strain erodes functioning while psychosocial resources promote resilience.^27^, ^28^

Social engagement moderated these associations in meaningful ways. Participants who regularly volunteered, attended religious services, or participated in community groups experienced a slower rate of cognitive decline even when reporting higher depressive symptoms or frequent restless sleep. The buffering effect was stronger for depressive symptoms, suggesting that engagement may provide emotional regulation, purpose, routine, and social affirmation that help counteract depressive states.^16^, ^29^, ^30^ Although the buffering effect for sleep disturbance was smaller in magnitude, it operated in the same direction and aligns with evidence that socially engaged individuals maintain more structured daily routines and experience reduced stress. These patterns are consistent with theoretical models of cognitive reserve and socioemotional selectivity, which propose that meaningful engagement supports neural efficiency and emotion regulation and helps individuals maintain cognitive functioning in the face of psychosocial risk.^18^, ^30^

These findings also have important cultural implications. For many older Black adults, religious, and community institutions serve as central sites of belonging, affirmation, social cohesion, and collective coping. Faith‐based involvement and community participation are historically rooted and culturally embedded practices that help buffer the long‐term effects of discrimination, economic hardship, and structural stressors.^1^, ^18^, ^30^ In this context, social engagement may function not only as a behavioral activity but as a culturally grounded resilience mechanism that protects cognitive health across the life course.

From a clinical and public health standpoint, these findings highlight the value of integrating social engagement into cognitive health promotion strategies. Encouraging participation in community, volunteer, or religious activities represents a low‐cost and non‐pharmacological approach to support cognitive aging. Health practitioners and community organizations can collaborate to identify socially isolated older adults and facilitate access to meaningful opportunities for engagement. Such initiatives could help slow cognitive decline while strengthening community connection and psychosocial well‐being.^21^, ^23^

## 5 LIMITATIONS AND CONCLUSION

Although this study employed longitudinal data and robust mixed‐effects modeling, several limitations should be noted. Depressive symptoms, restless sleep, and social engagement were measured only at baseline, which limits the ability to evaluate how changes in these psychosocial factors influence the rate of cognitive decline over time. Future research should incorporate time‐varying models that allow these predictors to fluctuate across waves. Additionally, sleep disturbance was assessed using a single self‐reported item, which may not fully capture the complexity of sleep quality. Objective measures such as actigraphy or polysomnography could strengthen future analyses. Despite adjusting for a wide range of covariates, unmeasured confounders, including early‐life adversity, neighborhood context, and biological stress markers may contribute to residual bias. Although the analytic sample was representative of older Black adults, generalizability to other populations should be made cautiously. Further work should explore the neurobiological and psychosocial pathways through which social engagement slows cognitive decline, including biomarkers of inflammation, chronic stress, and indicators of cognitive reserve. Longitudinal intervention studies designed to increase engagement through faith‐based, volunteer, or community programs would provide stronger evidence of causality.

In conclusion, this study provides novel longitudinal evidence that social engagement moderates the adverse effects of depressive symptoms and restless sleep on the rate of cognitive decline among older Black adults. The findings highlight social engagement as both a personal and community‐level mechanism of resilience that helps maintain cognitive functioning over time despite psychosocial risk. Strengthening opportunities for meaningful participation through culturally grounded, community‐based initiatives may represent a powerful strategy for advancing equitable cognitive aging in diverse populations.

## AUTHOR CONTRIBUTIONS

Darlingtina K. Esiaka and Sunkanmi Folorunsho contributed to the study's conception and design. Literature search was performed by Sunkanmi Folorunsho. Sunkanmi Folorunsho conducted data analysis, with input from Darlingtina K. Esiaka and Olufunmilola Abraham. The first draft of the manuscript was written by Sunkanmi Folorunsho, while Darlingtina K. Esiaka and Olufunmilola Abraham critically revised versions of the manuscript. All authors read and approved the final version of the manuscript for submission.

## CONFLICT OF INTEREST STATEMENT

The authors declare no conflicts of interest. Author disclosures are available in the supporting information.

## ETHICAL APPROVAL

Not applicable as the current study used publicly available dataset.

## CONSENT STATEMENT

This study uses secondary data from the Health and Retirement Study (HRS), which is sponsored by the National Institute on Aging (grant number U01AG009740) and conducted by the University of Michigan. The HRS obtains informed consent from all participants, and the de‐identified datasets are publicly available for research purposes. No additional institutional review board approval was required for this analysis.

## Supporting information



Supporting Information

**Supplementary Figure S1**. Individual cognitive trajectories (spaghetti plot) with mean trajectories overlaid for high and low social engagement.

## Data Availability

The data used in this study are from the Health and Retirement Study (HRS), sponsored by the National Institute on Aging (grant number U01AG009740) and conducted by the University of Michigan. Public‐use HRS datasets are freely available to registered users at https://hrs.isr.umich.edu/data‐products.
