# Supplementary figures and images for: Social engagement moderates the relationship between cognitive functioning, depressive symptoms, and restless sleep in older black adults
Source: Alzheimers Dement. 2026 Jan 12;22(1):e71078. doi: 10.1002/alz.71078 (PMC12796501; doi:10.1002/alz.71078)

### Supplementary Figure S1: Spaghetti plot

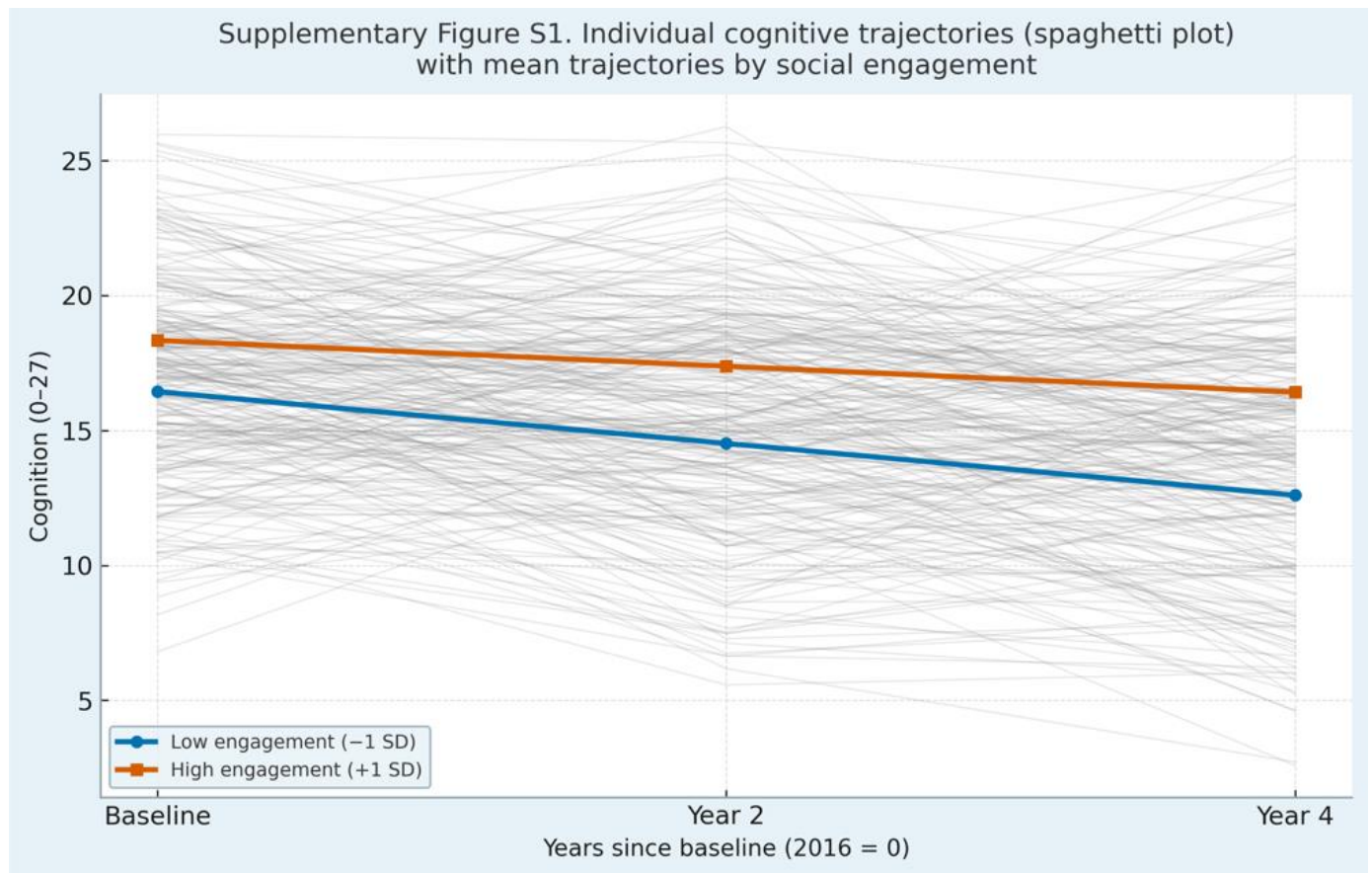

Supplement: Supplementary file 2 — Supplementary Figure S1. Individual cognitive trajectories (spaghetti plot) with mean trajectories overlaid for high and low social engagement. [file ALZ-22-e71078-s001.pdf]
